# Parkinson’s Disease and Frailty: A Two-Way Link Across Aging

**DOI:** 10.3390/jcm15010063

**Published:** 2025-12-22

**Authors:** Daniel Hernández-Triana, Salomón Páez-García, Alexandre Mena, Mar Gimeno, Alejandra Soto-Leal, Maria Cruz Rodriguez-Oroz, Miguel Germán Borda

**Affiliations:** 1 Semillero de Neurociencias y Envejecimiento, Pontificia Universidad Javeriana, Bogotá 110231, Colombia; danieruht@gmail.com (D.H.-T.); salomondavidpaez@gmail.com (S.P.-G.); alesotoleals@gmail.com (A.S.-L.); 2 Semillero de Neurociencias y Envejecimiento, Clínica Universidad de Navarra, 31008 Pamplona, Spain; amenaandres@alumni.unav.es; 3 Department of Neurology, Clínica Universidad de Navarra, 31008 Pamplona, Spain; mgimenor@unav.es (M.G.); mcroroz@unav.es (M.C.R.-O.); 4 Centre for Age-Related Medicine (SESAM), Stavanger University Hospital, Helse Stavanger HF, P.O. Box 8100, 4068 Stavanger, Norway

**Keywords:** Parkinson’s disease, frailty, aging biology, comprehensive geriatric assessment, exercise, nutrition, cognitive decline, outcomes

## Abstract

**Background:** Parkinson’s disease (PD) and frailty frequently co-occur and may interact bidirectionally through shared mechanisms of aging biology, mitochondrial dysfunction, inflammation, and reduced physiological reserve. **Objective:** We aimed to synthesize current evidence on prevalence, directionality, clinical overlap, adverse outcomes, and management implications of the PD–frailty nexus. **Methods:** A narrative review of epidemiologic, cohort, and interventional studies was performed, examining frailty in PD and PD risk in prefrail/frail populations, plus trials of multimodal interventions. **Results:** Frailty is common in PD, affecting approximately one-third of patients overall and becoming more prevalent as the disease advances. It independently predicts falls, cognitive decline, hospitalization, institutionalization, and mortality. Large cohorts suggest prefrailty/frailty is associated with incident PD risk, supporting a potential bidirectional association rather than direct causation. Diagnostic complexity arises because PD motor and non-motor features overlap with frailty constructs, risking misclassification. Management based on Comprehensive Geriatric Assessment (CGA) enhances personalized, multidisciplinary care. Exercise, particularly combined aerobic and resistance training reduces frailty and improves mobility, postural control, and quality of life. Complementary nutritional strategies, including muscle-targeted supplementation, can further strengthen rehabilitation outcomes, while careful attention to social determinants and polypharmacy remains essential to optimizing overall health and functional independence. **Conclusions:** Frailty is best understood as a clinical marker of vulnerability within PD and a correlate of more adverse trajectories rather than a proven causal determinant. Systematic frailty assessment integrated into PD care may help refine prognosis, individualize treatment, and support efforts to preserve independence. Priorities include PD-adapted frailty tools, CGA implementation, and rigorous trials of combined exercise–nutrition programs.

## 1. Introduction

Parkinson’s disease (PD) is a progressive neurodegenerative disorder with an estimated prevalence of approximately 1.5 cases per 1000 individuals, making it the second most prevalent neurodegenerative disorder worldwide, following Alzheimer’s disease [1]. It is characterized by gradual loss of dopaminergic neurons in the substantia nigra [2]. Pathologically, PD is marked by the accumulation of alpha-synuclein aggregates (known as Lewy bodies) and irreversible nigral dopaminergic cell loss [3]. Characteristic features of PD include motor symptoms such as tremors, bradykinesia, rigidity, and postural instability, which can result in difficulty maintaining balance and increased risk of falls. Additionally, non-motor symptoms are common and may include cognitive changes, mood disorders like depression and anxiety, sleep disturbances, and autonomic dysfunction, which can manifest as orthostatic hypotension and urinary issues [4,5].

Older adults with PD may experience a decrease in their physiological reserves when several conditions or symptoms especially those related with PD occur simultaneously, leading to frailty, which is a condition characterized by a decline in physiological reserves and increased vulnerability to stressors across multiple organ systems [6]. Frailty is clinically manifested by weight loss, fatigue, weakness and slow gait [7]. Beyond these clinical manifestations, frailty is also strongly rooted in biological-aging mechanisms such as mitochondrial dysfunction, chronic low-grade inflammation (“inflammaging”), and neuromuscular degeneration, which may contribute to a shared vulnerability in patients with PD [8]. PD and frailty are conditions that become more frequent with age and represent major threats to independence and quality of life in older populations [9,10].

Although PD and frailty have traditionally been studied separately, growing evidence indicates that they frequently overlap in clinical practice [7]. Frailty is highly prevalent among individuals with PD, with reported rates varying widely depending on the assessment tool and disease stage [11]. Those who exhibit both frailty and PD are generally older, have more severe disease phenotypes involving non-motor symptoms, postural instability and gait disorders, and require higher doses of dopaminergic medication [11].

Importantly, the relationship between PD and frailty appears to be bidirectional. Notably, frailty may function as a prodromal indicator of early neurodegeneration, or it may exist as a condition associated with PD via shared antecedents such as inflammatory mechanisms [8,12]. PD progression accelerates the development of frailty through motor decline, fatigue, autonomic dysfunction, and multimorbidity [13]. Conversely, frailty itself may be linked to the risk of developing PD or exacerbate its severity [14]. However, despite this growing recognition, the temporal directionality, shared mechanistic pathways, and clinical implications of this interaction remain poorly defined. In addition, conventional frailty constructs were not designed for PD populations, raising concerns regarding misclassification and lack of standardized assessment [15].

For these reasons, it is necessary to clarify how frailty influences PD onset and trajectory, identify the biological convergence between the two syndromes, and inform practical management strategies tailored to vulnerable older adults with PD. Improving understanding of this intersection is essential for optimizing outcomes, preventing loss of independence, and supporting healthy aging in PD. This narrative review aims to integrate recent findings across epidemiologic, mechanistic, and clinical domains to characterize the PD–frailty nexus and highlight its emerging relevance for personalized care.

## 2. Materials and Methods

This narrative review was designed to synthesize current knowledge on the epidemiological, clinical, and mechanistic links between PD and frailty. A comprehensive search was conducted until November 2025 in PubMed, EMBASE, Scopus, Web of Science, and Google Scholar. The strategy combined Medical Subject Headings (MeSH), Emtree terms, and free-text keywords including “Parkinson’s Disease,” “Frailty,” “Aging,” “Sarcopenia,” “Gait Disorders,” “Falls,” “Cognitive Impairment,” “Comprehensive Geriatric Assessment,” and “Outcomes.”

Reference lists of selected studies were additionally screened using a snowball approach to identify further relevant literature. We included observational studies assessing frailty prevalence and outcomes in PD, studies evaluating PD risk in prefrail or frail populations, clinical trials of multimodal interventions targeting frailty in PD, mechanistic research on shared pathophysiological pathways, and high-quality reviews. There were no language restrictions, and although no strict publication year limit was applied, studies published from 2015 onward were prioritized to ensure contemporary relevance. Two reviewers (S.P.-G., D.H.-T.) independently screened titles, abstracts, and full texts, resolving discrepancies by consensus. Studies without full-text access, abstract-only reports, and those not assessing frailty-related constructs in PD were excluded. A total of 92 studies meeting eligibility criteria were included in the final qualitative synthesis.

This review was designed as a narrative synthesis; no new quantitative meta-analysis was performed. Effect estimates such as pooled prevalence and hazard ratios are reported as presented in the original studies and previously published systematic reviews or meta-analyses. We did not recalculate or statistically pool outcomes across studies.

## 3. Epidemiology of Frailty and PD

Frailty is substantially more prevalent among individuals with PD than in the general older population [15]. A systematic review by Smith et al. (2019) found prevalence estimates ranging from 29% to 67% [16]. Additionally, McMillan et al. (2021) reported a pooled prevalence of 38% (95% CI 24–55%) using Fried’s Frailty Phenotype [17]. Lee et al. (2025) noted high variability in prevalence rates, with estimates reaching as high as 76% depending on the instrument used [11]. This variability reflects the lack of standardization in frailty measurement, as studies employ diverse constructs, ranging from deficit accumulation to sarcopenia-based definitions [18]. Tertiary care cohorts with older, more advanced cases typically report higher rates than community-based studies, and heterogeneity is further influenced by age, sex, and PD severity [19]. Despite methodological differences, evidence consistently shows that roughly one-third of PD patients are frail [17].

Comparative analyses highlight this overrepresentation, showing that frailty affects 35.6% of patients with PD, while only 5.2% of age-matched controls are affected (*p* < 0.001) [19], and de novo PD cases show higher odds of frailty (OR 6.7, *p* < 0.001) [20].

Differences between phenotype-based and deficit-accumulation models partly explain divergent prevalence estimates in PD. Phenotype-based tools such as Fried’s criteria rely heavily on motor performance (gait speed, weakness) [21], which are core PD manifestations, thereby inflating frailty classification in individuals with prominent motor disability [15]. In contrast, Frailty Index based approaches incorporate multimorbidity, disability, cognition, and mood, potentially capturing non-motor PD burden rather than intrinsic physiological vulnerability [22].

## 4. Pathophysiological Mechanisms Linking Frailty and PD

Frailty and PD frequently coexist, sharing pathophysiological foundations rooted in biological aging and cellular stress [7]. Aging is characterized by genomic instability, cellular senescence and stem cell exhaustion [23], all of which heighten susceptibility to neurodegeneration and diminish the body’s regenerative capacity [24]. Beyond aging alone, the combined effects of oxidative stress, mitochondrial dysfunction, neuroinflammation, and metabolic alterations further contribute to the onset and progression of both disorders [25]. These interconnected mechanisms create an environment in which genetic predisposition and environmental exposures intensify both neuronal and systemic decline [25].

PD hallmark pathological changes are aggravated by mitochondrial dysfunction, caused by a reduction in complex I activity within the electron transport chain, which leads to decreased ATP production and excessive accumulation of reactive oxygen species (ROS) [26]. This oxidative stress damages mitochondrial DNA and triggers apoptotic signaling cascades that lead to neuronal death. Mutations in PINK1, PARKIN, DJ-1, and LRRK2 further impair mitophagy, causing the accumulation of dysfunctional mitochondria and exacerbating oxidative damage [26,27]. On the other hand, age-related immune changes and inflammation play important roles in the pathogenesis of frailty [28]. Inflammaging is characterized by a chronic age-related low-grade inflammation, with elevated levels of Interleukin-1, Interleukin-6 and tumor necrosis factor alpha, accompanied by reduced levels of Interleukin-10 [29]. This imbalance is driven by monocyte activation, adipose tissue dysfunction, physical inactivity, and the accumulation of senescent cells [30]. Furthermore, evidence suggests that systemic inflammation can disrupt the blood–brain barrier (BBB), allowing peripheral proinflammatory cytokines and other inflammatory mediators to enter the central nervous system (CNS), which triggers microglial activation and neuroinflammation [31]. This chronic microglial reactivity exacerbates neuronal damage and accelerates neurodegeneration, as seen in diseases like PD, linking systemic immune dysregulation with central pathology [32,33]. Activated microglia release cytokines and reactive species that further damage neurons, creating a feedback loop of inflammation and neurodegeneration [31]. This mechanism is supported by evidence from autoimmune and neurodegenerative disease models, where systemic inflammation leads to neuroimmune activation and cognitive or motor deficits [34]. This systemic inflammation acts as a critical driver of CNS pathology by compromising BBB integrity and promoting sustained microglial activation, which accelerates dopaminergic neuron loss in PD and other neurodegenerative conditions [32].

Together, these shared biological pathways illustrate a bidirectional interplay in which frailty may exacerbate neurodegenerative vulnerability, while PD-related neurobiological changes accelerate systemic decline, ultimately reinforcing a self-perpetuating cycle of functional deterioration.

## 5. Frailty as a Risk Factor for Incident PD

Recent evidence indicates a strong association between frailty and the risk of developing PD [13], suggesting that frailty may serve as an early clinical marker or predisposing factor associated with diagnosis. In the UK Biobank (*n* ≈ 315,000), individuals classified as prefrail or frail had significantly higher risks of incident PD, with hazard ratios of 1.26 (95% CI, 1.15–1.39) and 1.87 (95% CI, 1.53–2.28), respectively, compared with robust participants, independent of genetic predisposition [7]. Across multiple cohorts, frailty emerges not merely as a correlate but as a stable early clinical signal associated with PD risk. Medicare data further showed that individuals with higher frailty index scores exhibit elevated PD risk up to five years before diagnosis [35].

In addition, the HELIAD study conducted in Greece by Ntanasi et al. (2021), found that frailty, assessed using the Fried index, was associated with prodromal PD markers such as REM sleep behavior disorder, constipation, hyposmia, and many more, indicating that frailty could possibly precede the neurodegenerative changes of PD [13].

For example, in a 2025 population-based study by Hussain et al., which utilized data from the SHARE cohort across 15 European countries, biomarkers of physical and mental health related to frailty were identified as predictors of PD risk. Participants’ self-reported fear of falling or experiencing falls in the past six months, as well as low grip strength, were significantly associated with an increased likelihood of a future PD diagnosis [36].

It is important to consider that some individuals classified as prefrail or frail in population-based studies may already be experiencing subtle motor and non-motor changes that reflect prodromal PD [13], meaning that early frailty may occasionally function as an early clinical manifestation rather than a separate risk factor for disease development.

## 6. Shared Risk Factors and Determinants

PD and frailty share a complex set of overlapping risk factors, including non-modifiable elements (age, sex, genetic risk) and modifiable lifestyle factors such as metabolic dysfunction and comorbidities [37]. Insulin resistance and metabolic syndrome drive chronic low-grade inflammation, oxidative stress, and mitochondrial dysfunction-processes implicated in both dopaminergic neuronal vulnerability and the muscle wasting seen in frailty [38,39]. For example, diabetes is consistently associated with an increased risk and faster progression of PD. Chohan et al. (2021) demonstrated that type 2 diabetes accelerates motor and cognitive decline and has a causal relationship with PD onset [40,41]. Furthermore, dyslipidemia, obesity, and hypertension exacerbate vascular injury, while comorbidities like cardiovascular disease, osteoporosis, and chronic kidney disease amplify vulnerability through systemic inflammation and polypharmacy [42,43].

Nutritional status and musculoskeletal health are equally critical. Malnutrition and vitamin D deficiency (defined generally as 25-hydroxivitamin D levels < 20 ng/mL, but with lack of consensus) are common in both conditions [44,45]. Vitamin D deficiency correlates with disease severity, progression, and fall risk, likely due to the nutrient’s neuroprotective effects and its important role in muscle physiology [44,46]. These deficits intersect with sarcopenia, which frequently coexists with PD [47]. Sarcopenia—characterized by reduced muscle mass, strength—substantially increases disability and fall risk [48]. Although results from vitamin D supplementation trials remain heterogeneous, active vitamin D analogs show potential to prevent or attenuate sarcopenia in vulnerable populations [49], and may modulate the immune dysfunction observed in both PD and frailty [44]. Taken together, vitamin D deficiency may contribute to PD through multiple interconnected pathways involving neuromuscular decline, fall risk, neurodegeneration, and immune imbalance [44].

Neuropsychiatric and cognitive factors also act as shared determinants. Depression, anxiety, apathy, and cognitive impairment are prevalent in both syndromes; these symptoms discourage movement and social engagement, perpetuating a deleterious cycle of physical and mental deterioration [14]. Additionally, sleep disorders, particularly RBD and excessive daytime sleepiness, are strong predictors of both frailty and PD progression [50].

Social and environmental vulnerabilities further compound risk. Low socioeconomic status, limited education, poverty, and social isolation are associated with reduced access to healthcare, poor diet quality, and delayed diagnosis [7]. Environmental exposures, including pesticides, heavy metals, air pollution, and rural living, are known PD risk factors and may also contribute to frailty via oxidative and inflammatory stress [12].

Likewise, sedentary behavior and low physical activity are robustly linked to higher risk for frailty and worse PD outcomes [51]. In individuals with PD, reduced activity levels often begin early, driven by bradykinesia, fatigue, fear of falling, and mood disturbances, and may create a self-perpetuating cycle of deconditioning that leads to sarcopenia and frailty [14]. Longitudinal studies show that sedentary time predicts faster motor progression, poorer balance, and diminished quality of life, even after adjusting for disease severity [7].

## 7. Clinical Overlap and Challenges in Diagnosis

The clinical manifestations of frailty and PD exhibit numerous overlapping characteristics, especially prevalent among geriatric populations, thereby presenting significant diagnostic challenges. The principal motor manifestations associated with PD are integral to the diagnostic criteria for frailty, as exemplified by the Fried physical phenotype [14]. Consequently, when a physician evaluates an older patient presenting with reduced walking speed, diminished strength, and fatigue, they must discern whether these findings arise from primary neurodegeneration, a multisystem frailty process, or a combination of both [52]. Systematic reviews and meta-analyses highlight that standard frailty measures were not developed with PD in mind and may therefore overestimate frailty prevalence in this population by conflating disease-specific motor impairments with systemic physiological decline [17,37].

This challenge is exacerbated by the intersection of sarcopenia and weight loss, prevalent phenomena in both PD and frailty [19]. In the context of PD, unintentional weight loss is often multifactorial, attributed to heightened energy expenditure due to tremors and dyskinesia, diminished caloric intake linked to dysphagia, or side effects of dopaminergic therapy [53]. In contrast, weight loss in the context of frailty typically reflects a primary loss of muscle mass (sarcopenia) and broader nutritional insufficiency [7,37]. When frailty and PD coexist, identifying the primary driver of weight loss becomes challenging. While advanced imaging, bioimpedance analysis, and formal nutritional assessments can help elucidate the underlying mechanisms, clinical and research settings often rely on simpler measures such as self-reported weight loss or changes in body mass index (BMI) [54,55,56]. However, self-reported weight loss and changes in BMI are insufficient to distinguish between the contributing causes [57,58]. This ambiguity has important clinical implications, as management strategies differ substantially. PD-related weight loss may respond to targeted dysphagia therapy or optimization of dopaminergic treatment [59,60], whereas frailty-related sarcopenia requires interventions such as resistance training and tailored nutritional supplementation [61].

The presence of certain non-motor symptoms in PD such as fatigue, cognitive slowing, depression, and autonomic dysfunction increase the diagnostic complexity of frailty. Fatigue and exhaustion, reported in up to 50% of individuals with PD, are also key features of frailty [37,62]. Similarly, cognitive impairment and mood disorders are prevalent in both conditions; systematic reviews indicate that cognitive impairment occurs in up to 45% of PD patients [63,64]. Studies support the use of objective measures such as the Timed Up and Go (TUG) test, gait speed, and handgrip dynamometry for assessing mobility and frailty in PD [10,65]. These instruments provide reliable and quantifiable metrics and are less susceptible to subjective bias than questionnaire-based tools [65,66]. Nonetheless, their reliability does not preclude important limitations in the context of PD. Because these tests capture motor slowing, postural instability and bradykinesia, which are core manifestations of PD, they may artificially inflate frailty scores by attributing disease-related motor impairment to frailty rather than underlying PD [14]. Thus, while objective performance measures remain valuable, their interpretation in PD requires caution and contextual clinical judgment [66].

Differentiating frailty-related slowness from PD requires careful clinical contextualization. Features such as dopaminergic responsiveness, marked asymmetry of motor symptoms, freezing of gait, and motor fluctuations favor underlying parkinsonism [67,68], while multisystem vulnerability, weight loss, sarcopenia, and reduced reserve following minor stressors are more suggestive of frailty [69]. In practice, combined neurological and geriatric assessment is often required to avoid misclassification and to guide appropriate intervention.

## 8. Adverse Outcomes Associated with Frailty in PD

The coexistence of PD and frailty represents a high-risk phenotype associated with adverse clinical outcomes across cognitive, mobility, and survival domains. A primary concern is the accelerated trajectory of neurodegeneration; frail PD patients exhibit significantly higher rates of cognitive decline and a substantially greater risk of developing dementia compared to their non frail peers [14,19,64]. This relationship is evident even in the early stages of the disease, where frailty correlates with reduced quality of life and non-motor severity [17,70]. For instance, Belvisi et al. (2021) reported that higher frailty scores are significantly associated with profound cognitive deficits [14].

A recent longitudinal study further proves this relationship: in a population-based cohort of 192 newly diagnosed PD patients followed over three years, Borda et al. (2022) observed that frailty was not only prevalent from the earliest stages of the disease but also a strong independent predictor of cognitive decline [20]. In addition, large population-based and prospective studies, including those from Latin America, corroborate these findings, linking PD and parkinsonism with both prevalent and incident frailty [71].

Hospital-related outcomes are also significantly worse in this population. The meta-analysis by McMillan et al. (2021) demonstrated that frailty in PD is associated with prolonged hospital stays, higher rates of complications such as delirium and psychosis, and increased inpatient mortality [17]. These patients are more frequently discharged to rehabilitation or long-term care facilities, reflecting the compounding effects of frailty on recovery and independence [14]. Beyond hospitalization, the coexistence of frailty and PD accelerates the trajectory of disability and loss of autonomy. Frail PD patients exhibit greater dependency in activities of daily living and are at higher risk of institutionalization, mirroring the additive burden of both syndromes [17].

**Figure 1:**
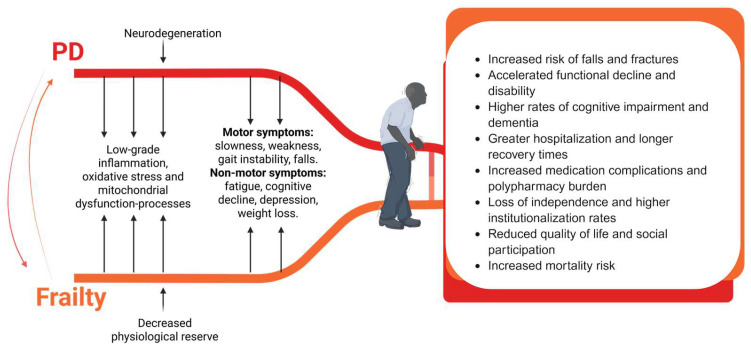
Conceptual model of the bidirectional relationship between PD and frailty. Shared mechanisms such as neurodegeneration, chronic low-grade inflammation, oxidative stress, and mitochondrial dysfunction contribute to decreased physiological reserve and worsening motor and non-motor symptoms when PD and frailty coexist. This interaction leads to a high-risk clinical phenotype characterized by increased falls, accelerated disability, cognitive impairment, more frequent hospitalizations, greater polypharmacy burden, reduced quality of life, and increased mortality risk. Created in BioRender. Borda, M. (2026) https://BioRender.com/w3d8ujj. Accessed on 30 October 2025.

Experimental evidence further supports these observations: Dallaire et al. (2024) demonstrated that frailty in PD is associated with a 70% larger sway area (A-COP, *p* = 0.013) and 23% higher sway velocity (VEL-AP, *p* = 0.038) compared with non-frail peers [72]. These alterations reflect reduced physiological reserve and poorer balance stability. Frail participants also had markedly lower grip strength (22.4 ± 6.3 kg vs. 31.4 ± 8.7 kg, *p* = 0.004), suggesting muscle weakness and fatigue as key contributors. These deficits translate into increased unstable gait, fear of falling, falls and diminished quality of life [72]. Complementary evidence associates frailty in PD with fatigue, orthostatic hypotension, and medication-related complications [17]. These interrelated impairments illustrate the cumulative burden of both syndromes on functional reserve and outcomes, as summarized schematically in Figure 1, which highlights how overlapping motor, non-motor, and systemic mechanisms drive a high-risk clinical trajectory in individuals with concurrent PD and frailty.

## 9. Clinical Implications and Management Strategies

Managing frailty in PD requires a personalized, multidisciplinary approach that incorporates proactive screening, CGA and collaborative care planning [22,37]. CGA plays a pivotal role as the organizing framework for the care of older adults with PD and frailty. It integrates multiple health domains such as clinical, functional, cognitive, psychological, nutritional, pharmacological, and social, into goal-directed care plans coordinated by interdisciplinary teams [37]. Referral to CGA is particularly recommended for individuals presenting with advanced age, functional decline, depression, weight loss, fatigue, or cognitive impairment [17]. During assessment, clinicians should consider the motor manifestations of PD and ensure multidisciplinary involvement to provide a holistic evaluation and care approach [64].

Currently, there are no universally accepted guidelines for managing frailty specifically in PD, and no frailty assessment tool has been validated for this population. Therefore, frailty scale scores must be interpreted within the clinical context to avoid misclassification and ensure individualized management. Despite these limitations, early identification of frailty remains essential for guiding treatment decisions and aligning interventions with realistic functional goals [17,22].

Weight loss is common in both conditions and often reflects a loss of both muscle and fat mass; as previously noted, it serves as a marker of poor outcomes [37]. Nutritional assessment in this population is key; recent reports show that muscle-targeted oral nutritional supplementation can improve muscle mass, strength, and physical performance in older adults with sarcopenia compared to placebo or standard care [73]. On the other hand, Barichella et al. (2019) conducted a randomized, assessor-blind controlled trial involving 150 patients with PD or parkinsonism undergoing 30 days of multidisciplinary intensive rehabilitation treatment [74]. They found that muscle-targeted nutritional support, consisting of a whey protein, leucine, and vitamin D-enriched supplement, led to a significantly greater improvement in functional performance, with an average 18 m higher gain in the 6 min walking test compared to a standard diet alone (*p* = 0.039) [74]. Nutraceuticals (such as polyphenols and omega-3 fatty acids) may function as adjunct therapies [75]. Antioxidant-rich formulations could limit levodopa-related oxidative stress in PD, potentially reducing side effects and prolonging treatment durability. Likewise, their antioxidant actions may potentiate exercise benefits, improving muscle function, cognitive health, and overall resilience, and thereby lowering frailty risk [75].

Multidisciplinary rehabilitation remains a cornerstone of frailty management in PD. Among available interventions, structured exercise programs are among the few strategies proven to reduce frailty and improve functional outcomes. Exercise interventions that combine aerobic and resistance training have been consistently linked to enhanced mobility, postural control, and quality of life in individuals with PD [17,22,37]. In addition, dual-task training and cueing strategies further support gait stability and motor performance, reinforcing the central role of multimodal, goal-oriented rehabilitation in comprehensive PD care [76]. A pilot study demonstrated that the introduction of daily physiotherapy and occupational therapy led to significant improvements in mobility and balance as reflected in metrics like the Barthel Index and the TUG test [77]. Innovative technologies, such as exoskeleton-assisted gait training, have shown measurable improvements in mobility [78]. Other approaches include Tai Chi, cycling, and axial biofeedback seem to have positive effects [37].

Despite the relevance of the social determinants of health, they are often overlooked in clinical care. These factors profoundly influence treatment adherence, rehabilitation engagement, nutritional status, and overall disease progression. Addressing them through integrated, person-centered care models and community-based support systems is essential to improving therapeutic outcomes and equity [79].

Polypharmacy is also a major concern—more than 40% of individuals with PD take five or more medications—and is associated with a 30–40% increased risk of developing frailty and functional decline [80,81]. This relationship is likely driven by the cumulative drug burden, particularly from anticholinergics, sedatives and antipsychotics, which are also linked to higher mortality and cognitively impaired older adults [82,83]. Although the specific medication classes most implicated in the PD–frailty interaction require further investigation, current management strategies emphasize careful medication review and deprescribing to mitigate frailty progression [84]. Regular medication reviews and minimizing anticholinergic burden using validated tools such as the Anticholinergic Cognitive Burden (ACB) and Anticholinergic Drug Scale (ADS) are recommended [85].

## 10. Discussion

This review synthesizes current evidence on the bidirectional relationship between PD and frailty, demonstrating that these two conditions frequently coexist and share overlapping biological, clinical, and prognostic domains. The convergence of aging biology mitochondrial dysfunction, chronic inflammation, and diminished physiological reserve provides a mechanistic foundation that explains why frailty is highly prevalent among patients with PD, affecting approximately a third of the population [16,17]. Rather than representing independent phenomena of aging, current evidence indicates that frailty modifies the expression of PD by accelerating vulnerability to stressors and diminishing functional reserve. This perspective highlights the need for clinicians to incorporate frailty evaluation into routine PD care, as recognizing vulnerability earlier may better inform prognosis and treatment planning. As this is a narrative synthesis without new pooled quantitative analyses, our findings should be interpreted as mainly descriptive and hypothesis-generating rather than providing definitive causal conclusions. By consolidating diverse epidemiologic, clinical, and mechanistic data, this work clarifies how frailty influences PD trajectories and emphasizes practical opportunities for earlier detection of vulnerability and more personalized care in real-world settings, although applicability may vary across populations.

Longitudinal evidence consistently shows that frailty in PD is not merely a comorbidity but a potent modifier of disease phenotype. As demonstrated by Borda et al. (2022), frailty predicts cognitive decline even in early-stage disease [20], while McMillan et al. (2021) confirmed its strong association with increased hospitalization, institutionalization, and mortality [17]. These findings shift the clinical focus from managing PD symptoms alone to targeting multisystem resilience. Furthermore, evidence from the UK Biobank indicates that frailty increases the risk of incident PD, reinforcing the concept that PD pathology is within a broader context of multisystem aging failure rather than as an isolated neurological event [7].

The clinical overlap between PD and frailty significantly complicates diagnosis and staging of both conditions. Features such as bradykinesia, weakness, slowed gait, weight loss and fatigue appear in both syndromes, challenging physicians to separate PD from frailty related functional decline. Dallaire et al. (2024) highlighted this by demonstrating that frail PD patients exhibit distinct postural deficits (e.g., increased sway area and lower grip strength) compared to non-frail peers [72]. This overlap has critical therapeutic implications: frail patients often have reduced tolerance to dopaminergic therapy, a higher burden of polypharmacy, and an increased risk of falls [20,80]. Consequently, standard aggressive pharmacological management might be less effective or less tolerated in this subgroup, necessitating a shift toward multimodal management. While interventional studies like Barichella et al. (2019) show that nutritional support and exercise can improve functional outcomes [74].

Exercise interventions for PD require a tailored approach distinct from general frailty treatments that primarily target sarcopenia through resistance training. PD specific evidence emphasizes the critical role of intensity dependent neuroplasticity and task specific training, with high intensity aerobic exercise and complex motor skill training shown to induce synaptic changes and improve functional reserve beyond simple muscle strengthening [86]. This suggests rehabilitation must address both systemic weakness and central motor network degradation [87]. Exercise promotes neuroplasticity by enhancing cortical-striatal plasticity and dopamine release, which may contribute to symptomatic relief and potential disease modification [88]. Overall, rehabilitation in PD should integrate high intensity aerobic and complex motor skill training to optimize functional outcomes by targeting both peripheral and central deficits.

Despite offering a consolidated view of the PD and frailty two-way link, this review has important limitations that must be acknowledged. The included studies exhibit considerable heterogeneity in frailty definitions and measurement instruments. As noted, prevalence estimates vary widely depending on whether the physical Frailty Phenotype or the Frailty Index is used, limiting comparability across studies and preventing robust meta-analyses [11]. These conceptual distinctions contribute to systematically higher frailty estimates when physical performance is emphasized and underscore the need for careful selection and interpretation of frailty tools in PD research and clinical assessment.

Additionally, most available evidence is observational, restricting causal inference. Although the biological hypothesis of bidirectional amplification is plausible, cross-sectional and short-term data do not allow a clear understanding of whether systemic dysregulation precedes neurodegeneration or vice versa.

Furthermore, many studies provide limited adjustment for key confounders and insufficient reporting on missing data, which weakens internal validity and limits the strength of inferences drawn from the available estimates. Moreover, as a narrative synthesis, this review did not include a formal risk-of-bias assessment, which may influence interpretation of study quality and strength of evidence.

Finally, there is limited longitudinal research integrating frailty trajectories with disease-specific biomarkers, leaving the mechanistic pathways underpinning this association only partially elucidated.

## 11. Future Directions

Future research should prioritize longitudinal, multidomain studies that integrate clinical phenotyping, biological aging markers, neuroimaging, and digital mobility metrics to clarify the temporal sequence linking frailty and PD. Harmonized frailty definitions and standardized assessment tools are essential to improve comparability across cohorts. Mechanistic studies exploring mitochondrial dysfunction, “inflammaging,” and synucleinopathy within frailty trajectories may reveal early intervention targets.

Crucially, the integration of wearable sensors and digital mobility markers represents a vital frontier assessment to continuously capture real world data on gait, turn velocity and physical activity pattern in PD. These technologies overcome the limitations of episodic clinical assessments by providing objective, sensitive endpoints that can monitor frailty progression more effectively [89,90]. Wearable sensor systems have been shown feasible for remote monitoring in PD, enabling frequent data collection outside clinical settings and supporting telemedicine interventions [91]. Although some studies found limited direct associations between specific gait parameters and quality of life in PD, continuous monitoring of mobility patterns remains valuable for tracking disease progression and motor fluctuations [92]. Altogether, wearable sensors and digital mobility markers represent a vital frontier for improving the assessment and management of frailty in PD through continuous, objective monitoring.

Additionally, interventional trials should evaluate whether addressing frailty through resistance training, nutritional optimization or anti-inflammatory strategies can modify PD progression and treatment response. Incorporating frailty assessment into PD clinical trials may also reduce heterogeneity in outcome interpretation, ensuring that therapeutic evaluations are precise. Finally, incorporating frailty screening and CGA into PD care could establish more effective management standards and promote personalized medicine that not only addresses symptoms but also preserves independence and quality of life.

## 12. Conclusions

Frailty and PD are closely related conditions that share common pathways. Rather than implying a direct causal relationship, current evidence suggests that frailty is associated with greater vulnerability and worse outcomes within the course of PD. Identifying frailty may help anticipate complications, support more accurate prognostic assessments, and guide care prioritization, although further research is needed to clarify its mechanistic role.

Regularly assessing frailty in PD patients is likely to help predict complications, customize treatments, and empower patients. Integrating geriatric principles into neurological care may shift management toward more preventive, resilience-focused strategies that preserve independence and quality of life. Longitudinal and interventional studies will be essential to determine whether addressing frailty can modify PD trajectories and improve long-term outcomes.

## Data Availability

The authors declare that there are no open-access data available in repositories. For any inquiries or requests related to the article, please contact the corresponding author.

## Abbreviations

The following abbreviations are used in this manuscript:

PD	Parkinson’s Disease
CNS	Central Nervous System
BBB	Blood–Brain Barrier
FI	Frailty Index
CI	Confidence Interval
OR	Odds Ratio
UK	United Kingdom
RBD	REM Sleep Behavior Disorder
ATP	Adenosine Triphosphate
ROS	Reactive Oxygen Species
PINK1	PTEN-Induced Kinase 1
LRRK2	Leucine-Rich Repeat Kinase 2
CGA	Comprehensive Geriatric Assessment
BMI	Body Mass Index
ACB	Anticholinergic Cognitive Burden
ADS	Anticholinergic Drug Scale
TUG	Timed Up and Go Test

## References

[1] Zhu J., Cui Y., Zhang J., Yan R., Su D., Zhao D., Wang A., Feng T. (2024). Temporal trends in the prevalence of Parkinson’s disease from 1980 to 2023: A systematic review and meta-analysis. Lancet Healthy Longev..

[2] Bloem B.R., Okun M.S., Klein C. (2021). Parkinson’s disease. Lancet.

[3] Saramowicz K., Siwecka N., Galita G., Kucharska-Lusina A., Rozpędek-Kamińska W., Majsterek I. (2023). Alpha-Synuclein Contribution to Neuronal and Glial Damage in Parkinson’s Disease. Int. J. Mol. Sci..

[4] Leite Silva A.B.R., Gonçalves De Oliveira R.W., Diógenes G.P., De Castro Aguiar M.F., Sallem C.C., Lima M.P.P., De Albuquerque Filho L.B., Peixoto De Medeiros S.D., Penido De Mendonça L.L., De Santiago Filho P.C. (2023). Premotor, nonmotor and motor symptoms of Parkinson’s Disease: A new clinical state of the art. Ageing Res. Rev..

[5] Páez-García S., Ramírez-Triana J., Folleco-Insuasty L., Orozco-Castro S., Fernández-de Castro S., Garcia-Cifuentes E., Cerquera-Cleves C. (2025). Non-motor symptoms in Parkinson’s disease: Recognition, diagnosis, and implications for comprehensive management. Acta Neurol. Colomb..

[6] Doody P., Lord J.M., Greig C.A., Whittaker A.C. (2023). Frailty: Pathophysiology, Theoretical and Operational Definition(s), Impact, Prevalence, Management and Prevention, in an Increasingly Economically Developed and Ageing World. Gerontology.

[7] Zheng Z., Lv Y., Rong S., Sun T., Chen L. (2023). Physical Frailty, Genetic Predisposition, and Incident Parkinson Disease. JAMA Neurol..

[8] Baechle J.J., Chen N., Makhijani P., Winer S., Furman D., Winer D.A. (2023). Chronic inflammation and the hallmarks of aging. Mol. Metab..

[9] Chhetri J.K., Mei S., Wang C., Chan P. (2023). New horizons in Parkinson’s disease in older populations. Age Ageing.

[10] Veronese N., Noale M., Cella A., Custodero C., Smith L., Barbagelata M., Maggi S., Barbagallo M., Sabbà C., Ferrucci L. (2022). Multidimensional frailty and quality of life: Data from the English Longitudinal Study of Ageing. Qual. Life Res..

[11] Lee J., Park J., Yoo S., Kim E., Yoo J.-H. (2025). Frailty in Patients with Parkinson’s Disease in Community and Clinical Settings: A Scoping Review. West. J. Nurs. Res..

[12] Tansey M.G., Wallings R.L., Houser M.C., Herrick M.K., Keating C.E., Joers V. (2022). Inflammation and immune dysfunction in Parkinson disease. Nat. Rev. Immunol..

[13] Ntanasi E., Maraki M., Yannakoulia M., Stamelou M., Xiromerisiou G., Kosmidis M.H., Dardiotis E., Hadjigeorgiou G., Sakka P., Gargalionis A. (2021). Frailty and Prodromal Parkinson’s Disease: Results from the HELIAD Study. J. Gerontol. Ser. A.

[14] Belvisi D., Canevelli M., Costanzo M., Giangrosso M., Fabbrini A., Borraccino A., Bruno G., Berardelli A., Fabbrini G. (2022). The role of frailty in Parkinson’s disease: A cross-sectional study. J. Neurol..

[15] Abraham D.S., Pham Nguyen T.P., Willis A.W. (2021). Claims-Based Frailty and Outcomes: Applying an Aging Measure to Older Adults with Parkinson’s Disease. Mov. Disord..

[16] Smith N., Brennan L., Gaunt D.M., Ben-Shlomo Y., Henderson E. (2019). Frailty in Parkinson’s Disease: A Systematic Review. J. Park. Dis..

[17] McMillan J.M., Michalchuk Q., Goodarzi Z. (2021). Frailty in Parkinson’s disease: A systematic review and meta-analysis. Clin. Park. Relat. Disord..

[18] Deng Y., Sato N. (2024). Global frailty screening tools: Review and application of frailty screening tools from 2001 to 2023. Intractable Rare Dis. Res..

[19] Peball M., Mahlknecht P., Werkmann M., Marini K., Murr F., Herzmann H., Stockner H., de Marzi R., Heim B., Djamshidian A. (2019). Prevalence and Associated Factors of Sarcopenia and Frailty in Parkinson’s Disease: A Cross-Sectional Study. Gerontology.

[20] Borda M.G., Pérez-Zepeda M.U., Jaramillo-Jimenez A., Chaudhuri K.R., Tovar-Rios D.A., Wallace L., Batzu L., Rockwood K., Tysnes O.-B., Aarsland D. (2022). Frailty in Parkinson’s disease and its association with early dementia: A longitudinal study. Park. Relat. Disord..

[21] Fried L.P., Tangen C.M., Walston J., Newman A.B., Hirsch C., Gottdiener J., Seeman T., Tracy R., Kop W.J., Burke G. (2001). Frailty in Older Adults: Evidence for a Phenotype. J. Gerontol. A. Biol. Sci. Med. Sci..

[22] Tenison E., Henderson E.J. (2020). Multimorbidity and Frailty: Tackling Complexity in Parkinson’s Disease. J. Park. Dis..

[23] Ma Y., Erb M.L., Moore D.J. (2025). Aging, cellular senescence and Parkinson’s disease. J. Park. Dis..

[24] Tan S.H., Karri V., Tay N.W.R., Chang K.H., Ah H.Y., Ng P.Q., Ho H.S., Keh H.W., Candasamy M. (2019). Emerging pathways to neurodegeneration: Dissecting the critical molecular mechanisms in Alzheimer’s disease, Parkinson’s disease. Biomed. Pharmacother..

[25] Coleman C., Martin I. (2022). Unraveling Parkinson’s Disease Neurodegeneration: Does Aging Hold the Clues?. J. Park. Dis..

[26] Meng K., Jia H., Hou X., Zhu Z., Lu Y., Feng Y., Feng J., Xia Y., Tan R., Cui F. (2025). Mitochondrial Dysfunction in Neurodegenerative Diseases: Mechanisms and Corresponding Therapeutic Strategies. Biomedicines.

[27] Tresse E., Marturia-Navarro J., Sew W.Q.G., Cisquella-Serra M., Jaberi E., Riera-Ponsati L., Fauerby N., Hu E., Kretz O., Aznar S. (2023). Mitochondrial DNA damage triggers spread of Parkinson’s disease-like pathology. Mol. Psychiatry.

[28] Tran Van Hoi E., De Glas N.A., Portielje J.E.A., Van Heemst D., Van Den Bos F., Jochems S.P., Mooijaart S.P. (2023). Biomarkers of the ageing immune system and their association with frailty–A systematic review. Exp. Gerontol..

[29] Coperchini F., Greco A., Teliti M., Croce L., Chytiris S., Magri F., Gaetano C., Rotondi M. (2025). Inflamm-ageing: How cytokines and nutrition shape the trajectory of ageing. Cytokine Growth Factor Rev..

[30] Wilson D., Jackson T., Sapey E., Lord J.M. (2017). Frailty and sarcopenia: The potential role of an aged immune system. Ageing Res. Rev..

[31] Millán Solano M.V., Salinas Lara C., Sánchez-Garibay C., Soto-Rojas L.O., Escobedo-Ávila I., Tena-Suck M.L., Ortíz-Butrón R., Choreño-Parra J.A., Romero-López J.P., Meléndez Camargo M.E. (2023). Effect of Systemic Inflammation in the CNS: A Silent History of Neuronal Damage. Int. J. Mol. Sci..

[32] Xu W., Huang Y., Zhou R. (2025). NLRP3 inflammasome in neuroinflammation and central nervous system diseases. Cell. Mol. Immunol..

[33] Fołta J., Rzepka Z., Wrześniok D. (2025). The Role of Inflammation in Neurodegenerative Diseases: Parkinson’s Disease, Alzheimer’s Disease, and Multiple Sclerosis. Int. J. Mol. Sci..

[34] Paroli M., Sirinian M.I. (2025). Pathogenic Crosstalk Between the Peripheral and Central Nervous System in Rheumatic Diseases: Emerging Evidence and Clinical Implications. Int. J. Mol. Sci..

[35] Karway G.K., Killion J.A., Faust I.M., Beyene K.M., Racette B.A., Camacho-Soto A. (2025). Frailty in prodromal Parkinson’s disease in Medicare beneficiaries. Park. Relat. Disord..

[36] Hussain M.A., Qaisar R., Karim A., Ahmad F., Franzese F., Alsaad S.M., Al-Masri A.A., Alkahtani S.A. (2024). Biomarkers of Physical and Mental Health for Prediction of Parkinson’s Disease: A Population-Based Study from 15 European Countries. Arch. Med. Res..

[37] Ebina J., Ebihara S., Kano O. (2022). Similarities, differences and overlaps between frailty and Parkinson’s disease. Geriatr. Gerontol. Int..

[38] Sian-Hulsmann J., Riederer P., Michel T.M. (2024). Metabolic Dysfunction in Parkinson’s Disease: Unraveling the Glucose–Lipid Connection. Biomedicines.

[39] Komici K., Pansini A., Bencivenga L., Rengo G., Pagano G., Guerra G. (2024). Frailty and Parkinson’s disease: The role of diabetes mellitus. Front. Med..

[40] Chohan H., Senkevich K., Patel R.K., Bestwick J.P., Jacobs B.M., Bandres Ciga S., Gan-Or Z., Noyce A.J. (2021). Type 2 Diabetes as a Determinant of Parkinson’s Disease Risk and Progression. Mov. Disord..

[41] Stockmann O., Ye L., Greten S., Chemodanow D., Wegner F., Klietz M. (2025). Impact of diabetes mellitus type two on incidence and progression of Parkinson’s disease: A systematic review of longitudinal patient cohorts. J. Neural Transm..

[42] Nussbaumerova B., Rosolova H. (2023). Obesity and Dyslipidemia. Curr. Atheroscler. Rep..

[43] Nicoletti A., Luca A., Baschi R., Cicero C.E., Mostile G., Davì M., La Bianca G., Restivo V., Zappia M., Monastero R. (2021). Vascular risk factors, white matter lesions and cognitive impairment in Parkinson’s disease: The PACOS longitudinal study. J. Neurol..

[44] Pignolo A., Mastrilli S., Davì C., Arnao V., Aridon P., Dos Santos Mendes F.A., Gagliardo C., D’Amelio M. (2022). Vitamin D and Parkinson’s Disease. Nutrients.

[45] Marcos-Pérez D., Sánchez-Flores M., Proietti S., Bonassi S., Costa S., Teixeira J.P., Fernández-Tajes J., Pásaro E., Valdiglesias V., Laffon B. (2020). Low Vitamin D Levels and Frailty Status in Older Adults: A Systematic Review and Meta-Analysis. Nutrients.

[46] Lv L., Tan X., Peng X., Bai R., Xiao Q., Zou T., Tan J., Zhang H., Wang C. (2020). The relationships of vitamin D, vitamin D receptor gene polymorphisms, and vitamin D supplementation with Parkinson’s disease. Transl. Neurodegener..

[47] Murphy K.T., Lynch G.S. (2023). Impaired skeletal muscle health in Parkinsonian syndromes: Clinical implications, mechanisms and potential treatments. J. Cachexia Sarcopenia Muscle.

[48] Gui M., Lv L., Hu S., Qin L., Wang C. (2025). Sarcopenia in Parkinson’s disease: From pathogenesis to interventions. Metabolism.

[49] Giustina A. (2024). Vitamin D at the crossroad of prediabetes, sarcopenia, and risk of falls. Lancet Healthy Longev..

[50] Aarsland D., Batzu L., Halliday G.M., Geurtsen G.J., Ballard C., Ray Chaudhuri K., Weintraub D. (2021). Parkinson disease-associated cognitive impairment. Nat. Rev. Dis. Primer.

[51] Padilha C., Souza R., Grossl F.S., Gauer A.P.M., De Sá C.A., Rodrigues-Junior S.A. (2023). Physical exercise and its effects on people with Parkinson’s disease: Umbrella review. PLoS ONE.

[52] Sargent L., Nalls M., Starkweather A., Hobgood S., Thompson H., Amella E.J., Singleton A. (2018). Shared biological pathways for frailty and cognitive impairment: A systematic review. Ageing Res. Rev..

[53] Sertpoyraz F.M., Korkmaz T., Çiftçi Y., Altaş E.U., Yıldırım E., Görmüş Saçan H.S. (2025). The Relationship of Sarcopenia with Disease Stage and Activities of Daily Living in Parkinson’s Patients. Med. Rec..

[54] Lima D.P., Monteiro P.A., Gomes De Luna J.R., Viana-Júnior A.B., Santos L.T.R., De Almeida S.B., Saldanha R.R.F., De Alencar M.S., Lopes F.K.D.M., Alencar Á.P. (2025). Assessment of body composition, sarcopenia and protein intake in mild to moderate Parkinson’s disease. Front. Nutr..

[55] Doganay M., Halil M.G., Kaymak C., Selek U., Topcuoglu M.A., Yalcin S. (2025). Expert opinion on the current conceptual, clinical and therapeutic aspects of disease related malnutrition and muscle loss: A multidisciplinary perspective. Front. Nutr..

[56] Tan A.H., Hew Y.C., Lim S.-Y., Ramli N.M., Kamaruzzaman S.B., Tan M.P., Grossmann M., Ang B.H., Tan J.Y., Manap M.A.A.A. (2018). Altered body composition, sarcopenia, frailty, and their clinico-biological correlates, in Parkinson’s disease. Park. Relat. Disord..

[57] Yilmaz M., Atik-Altinok Y., Seyidoglu Yüksel D., Acarer A., Bozkurt D., Savas S., Sarac Z.F., Akcicek F. (2025). Evaluation of sarcopenia and phase angle in elderly patients with Parkinson’s Disease. Int. J. Neurosci..

[58] Valent D., Peball M., Krismer F., Lanbach A., Zemann S., Horlings C., Poewe W., Seppi K. (2022). Different assessment tools to detect sarcopenia in patients with Parkinson’s disease. Front. Neurol..

[59] Artaud F., Lee P.-C., Mangone G., Vidailhet M., Corvol J.-C., Elbaz A. (2020). Longitudinal association between dopamine agonists and weight in Parkinson’s disease. Park. Relat. Disord..

[60] Bhidayasiri R., Phuenpathom W., Tan A.H., Leta V., Phumphid S., Chaudhuri K.R., Pal P.K. (2022). Management of dysphagia and gastroparesis in Parkinson’s disease in real-world clinical practice–Balancing pharmacological and non-pharmacological approaches. Front. Aging Neurosci..

[61] Millan-Domingo F., Garcia-Dominguez E., Gambini J., Olaso-Gonzalez G., Viña J., Gomez-Cabrera M.C. (2024). Diet and exercise in frailty and sarcopenia. Molecular aspects. Mol. Asp. Med..

[62] Siciliano M., Trojano L., Santangelo G., De Micco R., Tedeschi G., Tessitore A. (2018). Fatigue in Parkinson’s disease: A systematic review and meta-analysis. Mov. Disord..

[63] Peña-Zelayeta L., Delgado-Minjares K.M., Villegas-Rojas M.M., León-Arcia K., Santiago-Balmaseda A., Andrade-Guerrero J., Pérez-Segura I., Ortega-Robles E., Soto-Rojas L.O., Arias-Carrión O. (2025). Redefining Non-Motor Symptoms in Parkinson’s Disease. J. Pers. Med..

[64] Sousa-Fraguas M.C., Rodríguez-Fuentes G., Lastra-Barreira D., Conejo N.M. (2025). Associations between frailty and cognitive impairment in Parkinson’s disease: A cross-sectional study. Aging Clin. Exp. Res..

[65] Soares C.L.D.A., Ribeiro I.L., Benfica P.D.A.Y., Casado P.V., Swarowsky A., Faria C.D.C.D.M. (2025). Performance-based tests in individuals with Parkinson’s disease: Outcome scores and reliability. Clin. Rehabil..

[66] Sebastia-Amat S., Tortosa-Martínez J., Pueo B. (2025). Motor Assessment Timed Test (MATT): A New Timed Test to Assess Functional Mobility in Parkinson’s Disease Patients. J. Clin. Med..

[67] Kou W., Cai H., Cui Y., Zhu J., Li S., Yang C., Chen H., Feng T. (2025). Dopaminergic responsiveness and dopaminergic challenge tests of Parkinson’s disease: A systematic review and meta-analysis. J. Neurol..

[68] Zoetewei D., Ginis P., Herman T., Gilat M., D’Cruz N., Palmerini L., Gazit E., Hausdorff J.M., Nieuwboer A. (2025). The effects of dopaminergic medication and task load on trembling and akinetic freezing of gait in Parkinson’s disease. J. Neurol..

[69] Lynch D.H., Spangler H.B., Franz J.R., Krupenevich R.L., Kim H., Nissman D., Zhang J., Li Y.-Y., Sumner S., Batsis J.A. (2022). Multimodal Diagnostic Approaches to Advance Precision Medicine in Sarcopenia and Frailty. Nutrients.

[70] Pytel A., Beszlej J.A., Biercewicz M., Roszmann A., Krówczyńska D., Kołtuniuk A. (2022). The Effect of Frailty Syndrome on the Quality of Life of Individuals with Parkinson’s Disease: A Pilot Observational and Multicenter Study on the Polish Population. Int. J. Environ. Res. Public. Health.

[71] Kim D.J., Khan N., Llibre-Rodriguez J.J., Jiang M., Rodriguez-Salgado A.M., Acosta I., Sosa A.L., Acosta D., Jimenez-Velasquez I.Z., Guerra M. (2024). Cross-Sectional and Prospective Associations between Parkinsonism and Parkinson’s Disease with Frailty in Latin America. Mov. Disord. Clin. Pract..

[72] Dallaire M., Houde-Thibeault A., Bouchard-Tremblay J., Wotto E.A., Côté S., Santos Oliveira C., Ngomo S., Da Silva R.A. (2024). Impact of frailty and sex-related differences on postural control and gait in older adults with Parkinson’s Disease. Exp. Gerontol..

[73] Cereda E., Pisati R., Rondanelli M., Caccialanza R. (2022). Whey Protein, Leucine- and Vitamin-D-Enriched Oral Nutritional Supplementation for the Treatment of Sarcopenia. Nutrients.

[74] Barichella M., Cereda E., Pinelli G., Iorio L., Caroli D., Masiero I., Ferri V., Cassani E., Bolliri C., Caronni S. (2019). Muscle-targeted nutritional support for rehabilitation in patients with parkinsonian syndrome. Neurology.

[75] Montanari M., Mercuri N.B., Martella G. (2024). Exceeding the Limits with Nutraceuticals: Looking Towards Parkinson’s Disease and Frailty. Int. J. Mol. Sci..

[76] Zheng Y., Meng Z., Zhi X., Liang Z. (2021). Dual-task training to improve cognitive impairment and walking function in Parkinson’s disease patients: A brief review. Sports Med. Health Sci..

[77] Doyle M., Ryan D., Mello S. (2021). Efficacy of an elective period of in-patient rehabilitation for patients living with Parkinson’s Disease and Frailty: A pilot study. Age Ageing.

[78] Kotani N., Morishita T., Yatsugi A., Fujioka S., Kamada S., Shiota E., Tsuboi Y., Inoue T. (2020). Biofeedback Core Exercise Using Hybrid Assistive Limb for Physical Frailty Patients with or Without Parkinson’s Disease. Front. Neurol..

[79] Navarta-Sánchez M.V., Palmar-Santos A., Pedraz-Marcos A., Reidy C., Soilemezi D., Haahr A., Sørensen D., Smidt H.R., Bragstad L.K., Hjelle E.G. (2023). Perspectives of people with Parkinson’s disease and family carers about disease management in community settings: A cross-country qualitative study. J. Clin. Nurs..

[80] Bhagavathula A.S., Tesfaye W., Vidyasagar K., Fialova D. (2022). Polypharmacy and Hyperpolypharmacy in Older Individuals with Parkinson’s Disease: A Systematic Review and Meta-Analysis. Gerontology.

[81] Gutiérrez-Valencia M., Izquierdo M., Cesari M., Casas-Herrero Á., Inzitari M., Martínez-Velilla N. (2018). The relationship between frailty and polypharmacy in older people: A systematic review. Br. J. Clin. Pharmacol..

[82] Mach J., Gemikonakli G., Logan C., Vander Wyk B., Allore H., Ekambareshwar S., Kane A.E., Howlett S.E., De Cabo R., Le Couteur D.G. (2021). Chronic Polypharmacy with Increasing Drug Burden Index Exacerbates Frailty and Impairs Physical Function, with Effects Attenuated by Deprescribing, in Aged Mice. J. Gerontol. Ser. A.

[83] Porter B., Arthur A., Savva G.M. (2019). How do potentially inappropriate medications and polypharmacy affect mortality in frail and non-frail cognitively impaired older adults? A cohort study. BMJ Open.

[84] Ibrahim K., Cox N.J., Stevenson J.M., Lim S., Fraser S.D.S., Roberts H.C. (2021). A systematic review of the evidence for deprescribing interventions among older people living with frailty. BMC Geriatr..

[85] Nawaz H., Sargent L., Quilon H., Cloud L.J., Testa C.M., Snider J.D., Lageman S.K., Baron M.S., Berman B.D., Zimmerman K. (2022). Anticholinergic Medication Burden in Parkinson’s Disease Outpatients. J. Park. Dis..

[86] Zhen K., Zhang S., Tao X., Li G., Lv Y., Yu L. (2022). A systematic review and meta-analysis on effects of aerobic exercise in people with Parkinson’s disease. NPJ Park. Dis..

[87] Feng Y.-S., Yang S.-D., Tan Z.-X., Wang M.-M., Xing Y., Dong F., Zhang F. (2020). The benefits and mechanisms of exercise training for Parkinson’s disease. Life Sci..

[88] Chen X., Zhang G., Liu M., He J., Zhang Z. (2025). New perspectives on molecular mechanisms underlying exercise-induced benefits in Parkinson’s disease. NPJ Park. Dis..

[89] Adams J.L., Lizarraga K.J., Waddell E.M., Myers T.L., Jensen-Roberts S., Modica J.S., Schneider R.B. (2021). Digital Technology in Movement Disorders: Updates, Applications, and Challenges. Curr. Neurol. Neurosci. Rep..

[90] Bendig J., Wolf A.-S., Mark T., Frank A., Mathiebe J., Scheibe M., Müller G., Stahr M., Schmitt J., Reichmann H. (2022). Feasibility of a Multimodal Telemedical Intervention for Patients with Parkinson’s Disease—A Pilot Study. J. Clin. Med..

[91] Kheirkhahan M., Nair S., Davoudi A., Rashidi P., Wanigatunga A.A., Corbett D.B., Mendoza T., Manini T.M., Ranka S. (2019). A smartwatch-based framework for real-time and online assessment and mobility monitoring. J. Biomed. Inform..

[92] Sidoroff V., Raccagni C., Kaindlstorfer C., Eschlboeck S., Fanciulli A., Granata R., Eskofier B., Seppi K., Poewe W., Willeit J. (2021). Characterization of gait variability in multiple system atrophy and Parkinson’s disease. J. Neurol..

